# Identification of chilling and heat requirements of cherry trees—a statistical approach

**DOI:** 10.1007/s00484-012-0594-y

**Published:** 2012-10-06

**Authors:** Eike Luedeling, Achim Kunz, Michael M. Blanke

**Affiliations:** 1 World Agroforestry Centre, PO Box 30677-00100, Gigiri, Nairobi, 00100 Kenya; 2 Campus Klein-Altendorf, University of Bonn, Meckenheimer Str. 42, 53359 Rheinbach, Germany; 3 INRES—Horticultural Science, University of Bonn, 53121 Bonn, Germany

**Keywords:** Fruit trees, Dynamic model, Partial least squares regression, Projection-to-latent-structures regression, Climate change, Phenology, chillR

## Abstract

Most trees from temperate climates require the accumulation of winter chill and subsequent heat during their dormant phase to resume growth and initiate flowering in the following spring. Global warming could reduce chill and hence hamper the cultivation of high-chill species such as cherries. Yet determining chilling and heat requirements requires large-scale controlled-forcing experiments, and estimates are thus often unavailable. Where long-term phenology datasets exist, partial least squares (PLS) regression can be used as an alternative, to determine climatic requirements statistically. Bloom dates of cherry cv. ‘Schneiders späte Knorpelkirsche’ trees in Klein-Altendorf, Germany, from 24 growing seasons were correlated with 11-day running means of daily mean temperature. Based on the output of the PLS regression, five candidate chilling periods ranging in length from 17 to 102 days, and one forcing phase of 66 days were delineated. Among three common chill models used to quantify chill, the Dynamic Model showed the lowest variation in chill, indicating that it may be more accurate than the Utah and Chilling Hours Models. Based on the longest candidate chilling phase with the earliest starting date, cv. ‘Schneiders späte Knorpelkirsche’ cherries at Bonn exhibited a chilling requirement of 68.6 ± 5.7 chill portions (or 1,375 ± 178 chilling hours or 1,410 ± 238 Utah chill units) and a heat requirement of 3,473 ± 1,236 growing degree hours. Closer investigation of the distinct chilling phases detected by PLS regression could contribute to our understanding of dormancy processes and thus help fruit and nut growers identify suitable tree cultivars for a future in which static climatic conditions can no longer be assumed. All procedures used in this study were bundled in an R package (‘chillR’) and are provided as Supplementary materials. The procedure was also applied to leaf emergence dates of walnut (cv. ‘Payne’) at Davis, California.

**Electronic supplementary material:**

The online version of this article (doi:10.1007/s00484-012-0594-y) contains supplementary material, which is available to authorized users.

## Introduction

Recent climate change has had substantial impacts on the phenology of temperate plants, with many species showing advances in the timing of flowering in spring (Chmielewski and Rötzer 2001; Fitter and Fitter 2002; Menzel et al. 2006; Parmesan and Yohe 2003). This trend is commonly expected to continue into a future that is likely to be considerably warmer than recent decades (IPCC 2007). Yet some physiological characteristics of temperate plants make it uncertain that further warming will lead to further advances in phenology. Perennial plants of cold-winter climates fall dormant in winter in order to protect sensitive growing tissue from frost damage and to preserve nutrients assimilated over the previous season. To resume growth and initiate flowering in spring, they require winter chill (Erez 2000; Samish 1954; Vegis 1961; Campoy et al. 2011). Winter chill is an agroclimatic factor that integrates the length of cold periods as well as prevailing temperature ranges. It is thus a measure of ‘how long it has been how cold’. If chilling requirements are not met, irregular, delayed and asynchronous growth, flowering and fruit set are observed in the following growing season (Luedeling et al. 2009a; Campoy et al. 2011). One likely effect of climate change is a delay in the beginning of chill accumulation, the fulfillment of chilling requirements and thus the time at which trees become receptive to heat during spring. Since bloom and leaf emergence result from at least partially sequential fulfillments of cold (‘chilling’) and heat (‘forcing’) requirements, later and slower chilling accumulation should thus lead to later bloom and leafing in spring.

In fact, not all studies examining the response of temperate or boreal vegetation have found advances in phenology for all species analyzed. The studies of Fitter and Fitter (2002) and Menzel et al. (2006), which examined changes in the phenology of a large number of species, found advancing phenology for most species, but they also contained a sizeable proportion of species that showed stagnant or even delayed phenology over time, in spite of temperature increases. Recent studies have also shown delayed phenology for alpine grasslands on the Tibetan Plateau (Yu et al. 2010), vegetation in North America (Wang et al. 2011) and generally for plants at high northern latitudes (Delbart et al. 2006). There is thus evidence that warming can in fact delay spring phases. Yu et al. (2010) speculated that such delays could be related to plants’ vernalization requirements, and statistical evidence of a relationship between warm winters and late flowering has recently been produced for walnuts in California (Luedeling and Gassner 2012).

For growers of fruit and nut trees, which originate from temperate or relatively cool subtropical climates, selecting tree cultivars with appropriate chilling requirements for a given production site is critical for economically viable production. In order for cultivars of apple, cherry, pear, peach, almond, walnut, pomegranate, plum and many other species to be able to satisfy their cultivar-specific chilling requirements, production sites must have sufficiently cold and long winters. When the climate of a given production site warms, cultivar choices may have to be updated to avoid yield losses due to insufficient chilling. Horticultural scientists have developed several models for quantifying winter chill (e.g., Erez et al. 1990; Linsley-Noakes and Allan 1994; Richardson et al. 1974; Weinberger 1950), and these can be used in conjunction with climate projections to project future winter chill. In recent years, chilling losses have been projected for a range of locations, including California (Baldocchi and Wong 2008; Luedeling et al. 2009d), Australia (Darbyshire et al. 2011), Egypt (Farag et al. 2010) and high-mountain oases in Oman (Luedeling et al. 2009b). In contrast to these warm growing regions, an analysis of historic chilling trends in Germany showed little change over the last 50+ years (Luedeling et al. 2009a). A recent global analysis projected rather different trends for growing regions in different climates, with cold regions typically gaining winter chill, temperate regions seeing little change and warm regions experiencing severe losses (Luedeling et al. 2011). A comprehensive overview of studies on winter chill changes in response to climate change can be found in Luedeling (2012).

In light of projected changes in winter chill, many growers may have to transition to new cultivars in order to maintain satisfactory production levels. Such adaptation requires good understanding of current and projected future winter chill at production sites, and knowledge of the chilling requirements of commercially available tree cultivars. Both are currently not known for most places and for most cultivars. Even where estimates exist, they are often not directly useable, because they are frequently given in units that cannot be transferred easily to different locations (Luedeling and Brown 2011). Among fruit and nut producers, several models are in use for quantifying winter chill. In addition to a number of regional models (e.g., Gilreath and Buchanan 1981; Shaltout and Unrath 1983), three models are used widely around the world: the Chilling Hours Model (Bennett 1949; Weinberger 1950), the Utah Model (Richardson et al. 1974) and the Dynamic Model (Fishman et al. 1987a; Fishman et al. 1987b). The Chilling Hours Model is the oldest and simplest model, whereas the Dynamic Model is relatively recent and much more complex. Luedeling et al. (2009e) showed recently that these models respond very differently to climate change. Ratios between winter chill estimates calculated with the different models differ strongly around the globe (Luedeling and Brown 2011), as well as between years and sites within growing regions (Luedeling et al. 2009f). Using an appropriate and at least somewhat accurate model is critical for reliably matching cultivars with site-specific agroclimatic conditions. For a number of sites, chilling models have been compared, with most comparisons favoring the Dynamic Model over the others (e.g., Luedeling et al. 2009f; Ruiz et al. 2007; Zhang and Taylor 2011; Luedeling 2012). Yet even if an ideal model were adopted globally, problems would remain in quantifying a cultivar’s chilling requirements. This is because winter chill accumulation cannot be observed easily without experimentation and little is known about the time span during which trees accumulate winter chill.

This study aims to provide a new method for deriving chilling and forcing requirements of tree cultivars, which does not require experimentation but relies entirely on statistical analysis of long-term phenological records. While this method will not work for new cultivars or cultivars for which no phenology records exist, it can help improve projections of climate change impacts on well-established cultivars, on which many orchards around the world depend. Better insights into the timing of temperature responses of trees during the dormancy season are also provided and can be used to carry out in-depth physiological studies on plant processes during the indicated periods. Such studies may then contribute to closing of the knowledge gaps that currently constrain our ability to accurately predict bloom dates and project climate change impacts on orchards. Moreover, the method presented in this paper allows a rough comparison of the usefulness of the various chilling models that are in use, through evaluation of the consistency of calculated chilling and forcing requirements over long time periods. The objectives of this study were thus the application of partial least squares (PLS) regression for estimating agroclimatic requirements of cherries in Germany during the dormancy season (Luedeling and Gassner 2012), the development of easily interpretable illustrations of the timing of temperature responses, the comparison of three major chilling models and the provision of the method presented to the horticultural research community in an easy-to-use manner.

## Materials and methods

### Phenology data

Bloom dates of cherry trees (cv. ‘Schneiders späte Knorpelkirsche’) were recorded using the BBCH phenology scale (Meier et al. 1994) at Campus Klein-Altendorf (50.4°N; 6.99°E; 160 m a.s.l.)—the experimental station of the University of Bonn—between 1984 and 2008. The location is exposed to westerly Atlantic weather, tempered by the mild buffering climate of the Rhine valley to the east, resulting in an average yearly temperature of 9.8 °C with less than 600 mm annual rainfall (Blanke and Kunz 2009). The site is in the center of the Meckenheim fruit-growing region away from urban areas. Meckenheim is one of many typical European fruit-growing regions at very similar latitudes, including southwest England (Somerset), southeast England (Kent), The Netherlands, Belgium and several growing regions in Germany (Meckenheim, Rheinhessen and Dresden) as well as in Poland. Phenological flowering data were recorded at Klein-Altendorf from fully-grown, bearing cherry trees. New trees were planted successively to ensure that trees of appropriate age were available at all times. The dataset is complete, with the exception of 1985. The cultivar ‘Schneiders späte Knorpelkirsche’ is grown widely not only in this area, and has so far been well adapted to the local temperate climate. The cultivar was chosen because of its (1) widespread and growing popularity, (2) traditionally successful cultivation and high yields, and (3) alleged high chilling requirement.

### Weather data

Daily minimum and maximum temperatures have been recorded at Campus Klein-Altendorf since 1958 (Blanke and Kunz 2009). From this dataset, we extracted daily minimum and maximum temperatures for 1 July 1983–30 June 1984, as well as for 1 July 1985–30 June 2008. Out of the 8,767 days contained in these time spans, daily minimum temperatures for 11 days and daily maximum temperatures for 3 days were missing, corresponding to 0.13 and 0.03 % of minimum and maximum temperatures, respectively. These gaps were closed by linear interpolation. Mean daily temperatures were then computed as the arithmetic means between minimum and maximum temperatures.

Since chill and forcing models require hourly input data, such data were calculated from daily minimum and maximum temperatures, based on the latitude of Klein-Altendorf. Hourly data were produced using the idealized daily temperature curve proposed by Linvill (1989, 1990), with sunrise times, sunset times and day lengths computed after Spencer (1971) and Almorox et al. (2005).

### Identification of critical phases for cherry bloom

Temperatures were subjected to an 11-day running mean to ensure that phases in which temperature has important effects on dormancy progression are clearly recognizable in the outputs from statistical procedures (Luedeling and Gassner 2012). In this calculation, all daily temperature records are replaced by the mean temperature of a period starting 5 days before and ending 5 days after the respective date. All running mean temperatures were then assigned to dormancy seasons starting on 1 July and ending on 30 June of the following year. For leap years, the season already ended on 29 June. Results were correlated with cherry bloom dates observed at the end of the respective dormancy season. This process produced a dataset of 25 years of bloom data and smoothed daily mean temperature data during the preceding summer, fall and winter as well as current spring.

The data were analyzed by PLS regression (Luedeling and Gassner 2012; Wold et al. 2001). This method is used frequently for interpretation of hyperspectral remote sensing information (Min and Lee 2005; Luedeling et al. 2009c), because it handles highly autocorrelated data better than most other regression approaches, and can be used in situations where the number of independent variables substantially exceeds the number of dependent variables. Typical remote sensing applications have many similarities to establishing a relationship between observed phenological events and a much larger number of observed daily temperature variables. PLS regression has been shown recently to be useful in such situations (Luedeling and Gassner 2012; Yu et al. 2010). In this study, independent variables were 365 smoothed daily temperatures between the previous July and June. Dependent variables were bloom dates, expressed in Julian days (days of the year). In the PLS analysis, two latent factors were assumed to exist.

PLS analysis produces two major outputs. The variable-importance-in-the-projection (VIP) statistic indicates whether or not certain variables are important for explaining variation in the dependent variable. Values greater than 0.8 are commonly taken to signify importance (Wold 1995). The model coefficients of the centered and scaled data indicate the strength and direction of the effect. Negative coefficients imply that negative deviations of temperatures during the respective day are correlated negatively with the dependent variable. Positive coefficients signify the opposite. In the context of bloom dates, this means that, during phases where model coefficients are positive, high temperatures appear to delay bloom (lead to a greater Julian day number, meaning a later date). Where coefficients are negative, high temperatures seem to have a bloom-advancing effect. The absolute value of the model coefficient signifies the strength of the effect. The most relevant phases for explaining bloom dates are thus those phases that have VIP scores greater than 0.8 and high positive or negative model coefficients.

### Quantification of chilling and heat requirements

From the results of the PLS analysis, potential chilling and forcing phases were delineated. The chilling phase was identified by consistently positive and at least temporarily important (according to the VIP score) model coefficients. Positive model coefficients indicate that warm temperatures during the respective day delay bloom. This is consistent with the notion of a chilling requirement, according to which extraordinarily warm temperatures should delay the breaking of tree dormancy and thus lead to later bloom. The forcing phase, in contrast, was characterized by consistently important and negative model coefficients. Negative coefficients indicate that warm conditions advance bloom, meaning that the plant is receptive to heat in this phase. Such receptiveness is commonly assumed to result from fulfillment of the chilling requirement.

For the resulting chilling and forcing phases, start and end dates were extracted from the PLS results. These were not always unambiguous, so that different candidate dates were analyzed. Three different chilling models and one forcing model were then used to calculate chilling and forcing between all candidate dates. All models used in this study require hourly temperatures as input data, so that the hourly records described above were used for the subsequent analyses.

### Chilling models

Probably the most common chilling model, and one that is used widely, is the Chilling Hours Model, also known as the Weinberger Model (Bennett 1949; Weinberger 1950). This model, which was first developed for peaches in Georgia (United States), interprets all hours with temperatures between 0 and 7.2 °C as effective for chilling accumulation. These Chilling Hours are accumulated through the winter season.

The second model is the Utah Model (Richardson et al. 1974), which uses a weighting function to determine chilling effectiveness and accounts for an observed negative influence of high temperatures on winter chill accumulation. This model assigns no physiological effects to temperatures below 1.4 °C, a weight of 0.5 for temperatures between 1.4 and 2.4 °C and between 9.1 and 12.4 °C, a weight of 1 for temperatures between 2.4 and 9.1 °C, and negative weights of −0.5 for temperatures between 15.9 and 18 °C and of −1 for temperatures above 18 °C (Richardson et al. 1974; Luedeling et al. 2009e). This model is also used widely but, like the Chilling Hours Model, has been reported to perform poorly in warm climates.

The Dynamic Model, developed for fruit production in Israel (Fishman et al. 1987a, 1987b), is based on the assumption that winter chill accumulation results from a two-step process. In the first step, cool temperatures produce an intermediate chilling product, which can be destroyed by heat. The intermediate product can be converted into a permanent ‘Chill Portion’ in a process that is most efficient at moderate temperatures. These Chill Portions are summed up through the winter season. The equations that this model is based on are more complex than for the other models but have been described by several authors (Darbyshire et al. 2011; Luedeling and Brown 2011; Luedeling et al. 2009e). In model comparisons, the Dynamic Model typically performs equally well or better than the other models (Luedeling et al. 2009f; Ruiz et al. 2007; Zhang and Taylor 2011), leading several studies to recommend using it more widely (Luedeling et al. 2009f, 2011; Ruiz et al. 2007; Zhang and Taylor 2011; Perez et al. 2008; Erez 2000; Erez et al. 1990; Luedeling and Brown 2011).

### Forcing model

We used only one heat model in this study (Anderson et al. 1986). In this model, heat accumulation is based on three temperature estimates for physiological effects: the lower threshold for heat accumulation, the upper threshold and the critical temperature, above which heat is no longer effective. The equation (Anderson et al. 1986; Luedeling et al. 2009f) also includes a stress factor F, which was set to 1 in this study, because these cherry trees were grown in fertile soil with high nutrient content and water holding capacity. They should thus not have been subjected to particular stress. The lower threshold temperature was set to 4 °C, the upper threshold to 25 °C and the critical temperature to 36 °C, according to recommendations by Anderson et al. (1986).

### Statistical evaluation

Purely statistical approaches cannot produce definite estimates of chilling or heat requirements. Nevertheless, the present analysis can provide an indication of these plant needs. Assuming that chilling and forcing happen typically during the time windows indicated by the PLS analysis, and assuming further that the chilling and forcing models are appropriate for quantifying these climate factors, the mean chilling and forcing accumulations during the respective phases should approximate the crop requirements. These means were thus calculated. With respect to chilling models, variation of accumulated winter chill during the different phases also provides an indication of model quality. If chilling accumulation happens mostly during the time frame indicated by the PLS analysis, and chilling requirements are predetermined genetically, then one would expect the amount of winter chill that is accumulated in the time window to be fairly similar across years. We therefore calculated the standard deviations of chilling estimates for the different time periods for all three models. The model with the lowest standard deviation should be the most accurate among the models tested.

All analyses were implemented in the R 2.13.2 programming language (R Development Core Team 2011). An important part of the analysis was the contributed package ‘pls’ (Mevik et al. 2011). All new R functions were collected in an R package (‘chillR’), which is provided as Supplementary material to this article, along with a detailed tutorial of how to use the package.

### Transferability of the method

To evaluate the usefulness of PLS regression beyond the specific circumstances of the Klein-Altendorf cherry dataset, we also applied the method to a dataset of leaf emergence dates of walnut at Davis, California (described in detail in Luedeling and Gassner 2012; Luedeling et al. 2009f). Leaf emergence dates for the walnut cultivar ‘Payne’ were available for 54 years (since 1953), and daily temperatures were recorded at a nearby weather station. All steps of the analysis were similar to the procedure described above for the cherry dataset.

## Results

### Relevant phases for chill and forcing accumulation

**Fig. 1:**
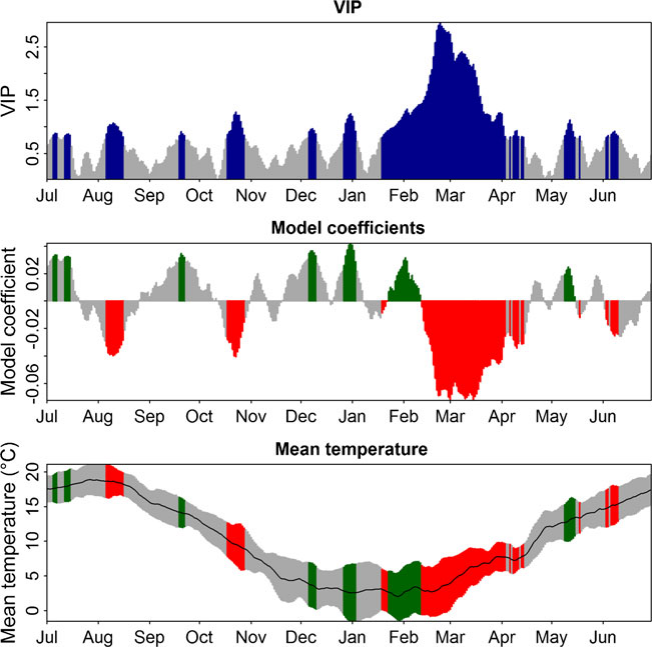
Results of Partial Least Squares (PLS) regression of bloom dates for cv. ‘Schneiders späte Knorpelkirsche’ cherries in Klein-Altendorf, Germany, with 11-day running means of daily mean temperatures. *Top panel* Variable importance in the projection (VIP), *middle panel* model coefficients of the centered and scaled data, *bottom panel* mean temperatures (*black line*) and their standard deviation (*grey areas*). *Blue bars* in the top panel indicate values above 0.8, the threshold for variable importance. In the middle and bottom figures, data for these dates is shown in *red* whenever model coefficients are negative, and *green* when they are positive

Several candidate periods for chill accumulation were identified. Focusing only on the fall and winter months, the VIP statistic of the PLS analysis showed important phases, during which model coefficients were positive, between 6 and 10 December, between 27 December and 3 January, and between 19 January and 11 February (Fig. 1). These phases were interrupted by periods during which model coefficients were mostly positive, but the VIP statistic did not identify them as important. Since trees are clearly dormant during this phase and probably accumulate winter chill, we considered the possibility that these phases were also effective for chilling accumulation. Candidate periods for the chilling phases were thus 1 November–11 February, 20 November–11 February, 20 November–21 December, 21 December–7 January and 23 January–11 February.

The forcing phase was more clearly recognizable in the output of the PLS analysis (Fig. 1). The model coefficients showed a large block of negative values between 12 February and 18 April. For all but 7 days during this period, VIP values were greater than 0.8, indicating importance. Much higher VIP scores up to 3.0 for this period compared to the chilling phases, with a maximum VIP of 1.4, indicate that temperature variation during the forcing phase has a stronger influence on bloom dates than variation during the chilling phases.

### Chilling and forcing requirements

Accumulated winter chill during the candidate chilling periods provides an approximation of the chilling requirements of cv. ‘Schneiders späte Knorpelkirsche’ cherry in Klein-Altendorf. Over all years for which bloom dates were available, mean accumulated winter chill during the longest of the candidate chilling periods (1 November–11 February) was 1,375 ± 178 Chilling Hours (mean ± standard deviation), 1,410 ± 238 Chill Units (Utah Model) and 68.6 ± 5.7 Chill Portions (Dynamic Model). For the second longest period (20 November–11 February), accumulated chill was 1,136 ± 164 Chilling Hours, 1,093 ± 227 Chill Units and 54.9 ± 5.4 Chill Portions. For the later potential chilling periods, chill totals were below 500 Chilling Hours, 500 Chill Units and 25 Chill Portions (Table 1). These seem unlikely to be the full chilling requirements of cv. ‘Schneiders späte Knorpelkirsche’ cherry, but the estimates may have relevance as partial requirements, in case different physiological processes have different chilling requirements that are fulfilled at different times. The shape of the PLS model coefficient graph indicates that up to three such periods may be occurring between the beginning of November and mid-February. Chilling requirements were defined consistently more clearly by the Dynamic Model, with coefficients of variation ranging from 8.3 % to 29.6 %, compared to 12.9 % to 37.9 % for the Chilling Hours Model and 16.9 % to 48.4 % for the Utah Model. In accordance with the principle that variation tends to be greater in small than in large datasets, the smallest coefficients of variation were found for the longest intervals examined. The smaller variation of chilling estimates around the mean in the Dynamic Model compared to the other models may indicate that this model is the most accurate among the models tested.

**Table 1:** Estimates of the chilling requirement of cv. ‘Schneiders späte Knorpelkirsche’ cherry in Klein-Altendorf, calculated for the Chilling Hours Model, the Utah Model and the Dynamic Model. Estimates are based on mean winter chill accumulated in the respective phases over 24 dormancy seasons, for which bloom dates were available

Start date	End date	Duration	Chilling hours model (Chilling Hours)		Utah model (Chill Units)		Dynamic model (Chill Portions)	
Day/month (Julian day)		Days	Mean ± SD	CV%	Mean ± SD	CV%	Mean ± SD	CV%
01/11 (306)	11/02 (42)	102	1,375 ± 178	12.9	1,410 ± 238	16.9	68.6 ± 5.7	8.3
20/11 (325)	11/02 (42)	83	1,136 ± 164	14.4	1,093 ± 227	20.8	54.9 ± 5.4	9.8
20/11 (325)	21/12 (356)	32	468 ± 79	16.9	446 ± 106	23.8	21.8 ± 1.6	7.3
21/12 (356)	07/01 (7)	17	224 ± 85	37.9	221 ± 107	48.4	10.8 ± 3.2	29.6
23/01 (23)	11/02 (42)	20	259 ± 86	33.2	238 ± 113	47.5	12.9 ± 2.8	21.7

For the forcing phase, only one time window was characterized by consistently negative model coefficients and high VIP scores (Table 2). For this phase, from 12 February to 18 April, 3,473 ± 1,236 growing degree hours were accumulated. The coefficient of variation was relatively high at 35.6 %, indicating either that the heat requirement is not a fixed value, that the forcing model is not very accurate, or that the start and end dates of heat accumulation differ strongly from year to year.

**Table 2:** Estimate of the heat (forcing) requirement (subsequent to chilling) of cv.’ Schneiders’ cherry in Klein-Altendorf, calculated after Anderson et al. (1986). Requirements were derived by summation of forcing units over the relevant period delineated by partial least squares (PLS) regression

Start date	End date	Duration	Forcing (growing degree hours)	
Day/month (Julian day)		Days	Mean ± SD	CV%
12/02 (43)	18/04 (108)	66	3,473 ± 1,236	35.6

### Transferability of the method

**Fig. 2:**
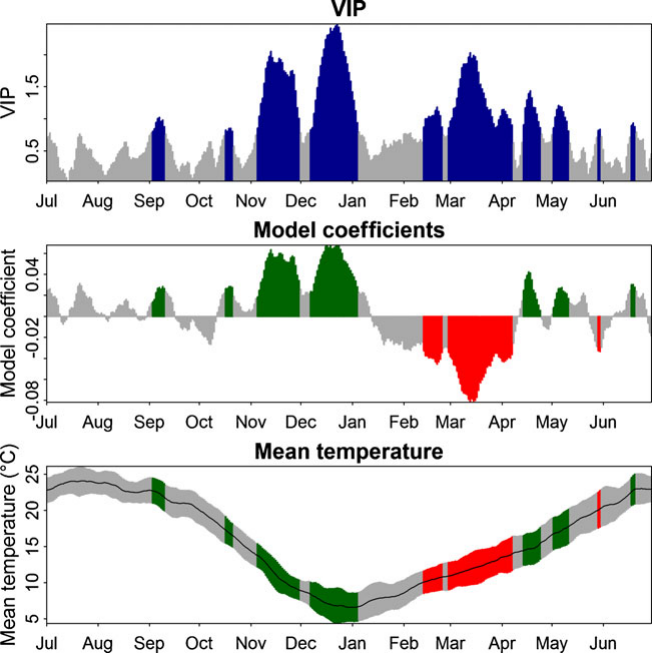
Results of the PLS regression of bloom dates for cv. ‘Payne’ walnuts at Davis, California, with 11-day running means of daily mean temperatures. *Top panel* VIP, *middle panel* model coefficients of the centered and scaled data, *bottom panel* mean temperatures (*black line*) and their standard deviation (*grey areas*). *Blue bars* in the top panel indicate values above 0.8, the threshold for variable importance. In the middle and bottom figures, data for these dates is shown in *red* whenever model coefficients are negative, and *green* when they are positive

Also in the output of PLS regression from analysis of the California walnut dataset, chilling and forcing phases were apparent (Fig. 2, Table 3). Probably due to the length of the observational record, phases were more distinct than in the dataset from Germany. Only two candidate phases for chill accumulation were identified (31 August–12 January and 12 October–12 January), and only one plausible phase of predominant forcing effects (13 Jan–10 April).

**Table 3:**
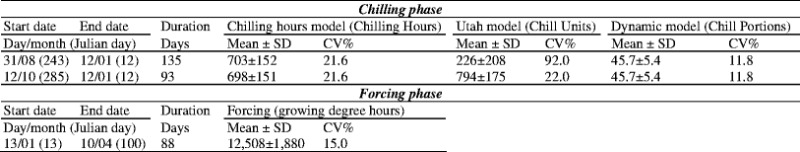
Estimates of the chilling and forcing requirement of cv. ‘Payne’ walnuts at Davis, California, for leaf emergence. Chilling requirements are calculated for the Chilling Hours Model, the Utah Model and the Dynamic Model and forcing requirements according to the growing degree hours concept. Estimates are based on mean winter chill and forcing accumulated in the respective phases over 54 dormancy seasons, for which leaf emergence dates were available

Based on these phases, chilling and forcing requirements for leaf emergence of cv. ‘Payne’ walnuts at Davis, California, were estimated at 45.7 Chill Portions (for both candidate periods) and 12,508 growing degree hours (Table 3). Estimates of chill accumulated during the two indicated phases were more consistent for the Dynamic Model (coefficient of variation of 11.8 % and 11.8 %) than for the Chilling Hours Model (21.6 % and 21.6 %) and the Utah Model (92.0 % and 22.0 %).

## Discussion

Phases during the winter that are likely to be relevant for accumulating chilling and heat became apparent in the PLS output, allowing approximate delineation of these potential phases based solely on statistics. Wherever long-term weather and phenology records are available, the method presented here makes determining these phases much easier than traditional approaches, which rely primarily on experimentation. It became clear from the PLS output, however, that the forcing phase was much more clearly defined and less ambiguous than the potential chilling phases. One reason for this may be that forcing is currently a much stronger driver of tree phenology in spring than chilling, in line with previous studies that have attributed historic changes in bloom dates almost exclusively to changes in spring heat (Chmielewski and Rötzer 2001; Fitter and Fitter 2002; Menzel et al. 2006; Parmesan and Yohe 2003). However, a previous study using PLS regression for illustrating dormancy progression of walnuts in California showed chilling phases more clearly (Luedeling and Gassner 2012; also shown in Fig. 2), indicating that PLS can provide a better delineation than seen here. It seems likely that the clarity of the chilling pattern in the PLS output is related to the winter temperature range at the study site. PLS regression identifies only the effects of deviations from ‘normal’ temperature patterns rather than effects of absolute temperatures. In California, where winter temperatures in the Central Valley rarely fall below freezing, positive deviations from the normal range typically mean that temperatures become suboptimal for chilling. This results in a clear PLS signature of positive model coefficients. In Klein-Altendorf, winters are much colder than in California, and frost is a common occurrence. According to all three commonly used chilling models, freezing temperatures are not effective for chilling, meaning that temperature increases may actually accelerate accumulation of winter chill, effectively advancing rather than delaying bloom dates (Luedeling and Brown 2011). In light of this fundamental difficulty of using the PLS method in cold climates, it is remarkable that chilling phases were visible nonetheless.

Most of the chilling phases identified by PLS regression occurred after early December. As an essential requirement of cherry bloom, these phases are at odds with the German custom of the ‘Barbara shoots’. According to this tradition, cherry shoots cut on 4 December (‘Saint Barbara’s Day’) bloom on Christmas Day, when forced indoors in a vase. This clearly shows that buds are able to bloom very early in the ‘chilling season’. It apparently conflicts with the common horticultural concept of high chilling requirements of cherries and with results presented in the present study. A general concept of chilling and forcing requirements of temperate trees derived by Harrington et al. (2010) from work on Douglas Fir in the Pacific Northwest, offers a potential explanation. Their concept does not treat chilling and forcing requirements as constants, but postulates that low chilling can be compensated by high forcing. Bud break occurs anywhere along a ‘possibility line’, at different combinations of chilling and forcing requirements. In this concept, a bud removed from chilling conditions on 4 December should require substantially more heat than a bud exposed to outside conditions throughout the entire winter. Indeed, assuming a constant room temperature of 15–20 °C, cherry shoots would accumulate between 5,500 and 10,000 growing degree hours for forcing between 4 and 24 December, substantially more than the 3,500 growing degree hours indicated by the present analysis. If the concept by Harrington et al. (2010) is valid for cv. ‘Schneiders späte Knorpelkirsche’ cherry in Germany, later chilling phases can be explained. This concept also indicates that trees may be more tolerant to insufficient chilling than is typically assumed, provided that more heat is available in spring. Before relying on this natural flexibility for adaptation, however, horticultural researchers should explore the possible physiological effects of suboptimal chilling/forcing combinations, which may have crop yield or quality implications.

In the output of the PLS analysis, the lack of a homogeneous phase of positive model coefficients with high VIP scores is probably because the effects of warm conditions during the winter on bloom dates are a mixture of bloom-delaying effects in exceptionally warm winter periods and bloom-advancing effects in cold winters. The short phase of important advancing effects of warming in mid-January seems likely to arise from a prevalence of cold conditions during that phase rather than signifying an interruption of the chilling phase. Much of the remaining variation in model coefficients and VIP scores can probably be explained by the balance between the two contrary effects of warming. Nevertheless, distinct phases of chilling effectiveness also appeared in the analysis of walnuts in California (Luedeling and Gassner 2012), where temperatures rarely fall to levels at which warming should meaningfully accelerate chilling accumulation. It may thus be worth considering the existence of qualitatively different phases of chilling accumulation in different periods of the dormancy progression. Rinne et al. (2011) recently produced genetic evidence of such different phases for *Populus*. They reported that winter chill induced the production of two substances that are important for breaking plant dormancy: (1) the peptide FLOWERING LOCUS T—a long-distance signal involved in breaking dormancy at the shoot apex; and (2) gibberellins, which are instrumental for reopening conduits in the embryonic shoots. If these processes are induced by different temperatures or occur at different times, this may well result in varying chilling effectiveness throughout the season, providing a possible explanation for the patterns found in the present study and by Luedeling and Gassner (2012).

The findings by Rinne et al. (2011) indicate that the chilling requirement of a cultivar may be composed of separate requirements for different processes, occurring at least partially at different times. The different candidate chilling periods identified based on the PLS output could provide an approximation of these, yet this is currently too speculative to merit further discussion. For quantifying the total chilling requirement of ‘Schneiders späte Knorpelkirsche’ cherries, which is the most relevant measure for growers, the longest (starting earliest and ending last) of the candidate periods seems like the most appropriate basis, so we base further discussions on this period. It is noteworthy that the start of the selected chill phase on 1 November coincides approximately with the beginning of leaf drop. Average dates of the beginning of leaf drop for cv. ‘Schneiders späte Knorpelkirsche’ occurred on 25 October, indicating this phenological stage as a possible signal for the cherry trees to start their chill accumulation.

Chilling totals between 1 November and 11 February varied over the years for all models, but were much more clearly defined for the Dynamic Model than for the other two models, according to coefficients of variation of 8.3 % for the Dynamic Model, compared to 12.9 % for the Chilling Hours Model and 16.9 % for the Utah Model. While this variation may simply reflect variation in winter chill, we find it likely that the lower variation in the Dynamic Model is also an indication of the latter model’s higher accuracy. This finding supports earlier research showing the Dynamic Model to perform equally well or better than the other models (Luedeling et al. 2009f; Ruiz et al. 2007; Zhang and Taylor 2011), and it adds weight to several studies that have recommended using it more widely (Luedeling et al. 2009f, 2011; Ruiz et al. 2007; Zhang and Taylor 2011; Perez et al. 2008; Erez 2000; Erez et al. 1990; Luedeling and Brown 2011). Results from the analysis of walnuts in California (Table 3) also show the most clearly defined chilling requirements for the Dynamic Model, adding further evidence to the finding that Chill Portions are a more useful metric for winter chill than the traditional measures.

According to the Dynamic Model, the chilling requirement of ‘Schneiders späte Knorpelkirsche’ cherry is 68.8 Chill Portions. Our calculated value is greater than estimates presented by Alburquerque et al. (2008) for sweet cherry cultivars in Murcia, Spain, for which they reported chilling requirements of between 30.4 and 57.6 Chill Portions based on 2 years of observation. Given that Klein-Altendorf has substantially colder winters than Murcia, a higher chilling requirement of a local cultivar is not an unexpected finding. The result is in line with the classification of cv. ‘Schneiders späte Knorpelkirsche’ as a high chill variety (ca. 1,500 chill hours). For cv. ‘Payne’ walnuts in California, a chilling requirement of 45.7 Chill Portions was determined. This value is quite different from the 66.1 Chill Portions indicated by Luedeling et al. (2009f) based on an analysis of the same dataset. This contrast highlights the importance of restricting the evaluation of chilling requirements to phases that are relevant for chill accumulation. The earlier study considered a much longer chilling phase than the one indicated by PLS regression, producing an estimate that was likely too high and contrasted with the common designation of ‘Payne’ as a low-chill cultivar.

We hope that the method presented in this paper will find several applications. Firstly, the estimates of chilling requirements and chilling phases obtained with this approach should benefit growers trying to identify appropriate cultivars suitable for their location. Of course, this will require widespread application of this method and the generation of a database with cultivar-specific chilling and heat requirements. Secondly, knowledge of the stages during the dormant phase when trees are responsive to temperature stimuli can help in the development of new rest-breaking strategies, e.g., by manipulating orchard climate (Campoy et al. 2010; Erez 1995) or applying plant growth regulators (de Salvador and di Tommaso 2003; Erez et al. 2008). Finally, our method can help guide research into dormancy processes, which is still a long way from full functional understanding. PLS regression can pinpoint the phases during which important temperature effects on plant physiology occur, and it can thus guide experimental work by geneticists and physiologists. The latter can then examine what happens during the indicated phases and hopefully help close the knowledge gaps that currently constrain the development of practical dormancy management options. Such options are urgently needed, in particular by producers in warm growing regions.

## Conclusions

PLS regression proved useful for analyzing long-term phenological records of cherry bloom at Klein-Altendorf (Germany), and also provided meaningful results for walnuts at Davis (California). The delineated chilling and forcing phases provided an indication of when these phases occur during the dormancy season. Determining these periods has thus far required experimentation, making this statistical approach a useful new tool in dormancy evaluation. It also allows a rapid appraisal of chilling requirements that could be useful for identifying tree cultivars that are suitable for a particular growing region now or in a future affected by climate change. It should be mentioned that this approach is applicable only where long-term observations of plant growth phases as well as good weather data are available. We estimate that at least 15 years of records are needed for producing reliable values for chilling and forcing requirements. Systematic collection of phenology and weather data, which currently does not happen in many places, can contribute significantly to closing the data gaps that currently constrain quantitatively appropriate adaptation of fruit and nut growers to climate change.

## Electronic supplementary material

Below is the link to the electronic supplementary material.

ESM 1 (PDF 128 kb)

ESM 2 (PDF 282 kb)

ESM 3 (GZ 36.4 kb)
