# Supplementary material for: Identification of chilling and heat requirements of cherry trees—a statistical approach
Source: Int J Biometeorol. 2012 Oct 6;57(5):679–89. doi: 10.1007/s00484-012-0594-y (PMC3745618; doi:10.1007/s00484-012-0594-y)
Supplement: Supplementary file 1 — (PDF 128 kb) [file 484_2012_594_MOESM1_ESM.pdf]

# Package ‘chillR’

September 27, 2012

**Type** Package

**Title** chillR

**Version** 0.5

**Date** 2012-08-16

**Author** Eike Luedeling

**Maintainer** Eike Luedeling <e.luedeling@cgiar.org>

**Description** This package calculates winter chill for temperate fruit trees with several common models from daily weather records. It also includes some functions to prepare weather records for such calculations and a function to statistically determine important temperature effects during the dormancy season based on long-term tree phenology records. These can be used to estimate chilling and forcing requirements of temperate fruit trees.

**Depends** pls

**License** GPL-3

**LazyLoad** yes

## R topics documented:

chillR-package . . . . . . . . . . . . . . . . . . . . . . . . . . . . . . . . . . . . 2
chilling . . . . . . . . . . . . . . . . . . . . . . . . . . . . . . . . . . . . . . . 4
interpolate_gaps . . . . . . . . . . . . . . . . . . . . . . . . . . . . . . . . . . . 6
KA_bloom . . . . . . . . . . . . . . . . . . . . . . . . . . . . . . . . . . . . . . 7
KA_weather . . . . . . . . . . . . . . . . . . . . . . . . . . . . . . . . . . . . . 7
make_hourly_temps . . . . . . . . . . . . . . . . . . . . . . . . . . . . . . . . . 8
PLS_pheno . . . . . . . . . . . . . . . . . . . . . . . . . . . . . . . . . . . . . . 9
stack_hourly_temps . . . . . . . . . . . . . . . . . . . . . . . . . . . . . . . . . 11

**Index** **13**

`chillR-package` *chillR*

## Description

This package calculates winter chill for temperate fruit trees with several common models from daily weather records. It also includes some functions to prepare weather records for such calculations and a function to statistically determine important temperature effects during the dormancy season based on long-term tree phenology records. These can be used to estimate chilling and forcing requirements of temperate fruit trees.

## Details

| | |
|---|---|
| Package: | chillR |
| Type: | Package |
| Version: | 0.5 |
| Date: | 2012-08-16 |
| Depends: | pls |
| License: | GPL-3 |
| LazyLoad: | yes |

The main purpose of this package is the statistical determination of chilling and forcing requirements of temperature fruit and nut trees based on long-term phenological datasets. The importance of such calculations is explored in detail in Luedeling (2012). The core of the procedure, which is documented in Luedeling et al. (2012 or 2013, in press in September 2012), is the function PLS_pheno, which conducts a PLS regression analysis on observed phenology data. These are correlated with running means of daily mean temperatures, derived from daily minimum and maximum temperatures. More details of the procedure are given in Luedeling and Gassner (2012). Based on (currently manual) interpretation of outputs from the PLS procedure, chilling and forcing requirements can be derived. This can be done using the chilling function of the package, which calculates chill during specified periods for all years included in the temperature records. The remaining functions interpolate_gaps, make_hourly_temps and stack_hourly_temps are needed for preparing temperature data for use in the analysis. Finally, the package includes the datasets KA_bloom and KA_weather. These are truncated versions of the data used to produce the results reported in Luedeling et al. (2012 or 2013).

## Author(s)

Eike Luedeling

Maintainer: Eike Luedeling <e.luedeling@cgiar.org>

## References

Luedeling E, Kunz A and Blanke M. Identification of chilling and heat requirements of cherry trees - a statistical approach. International Journal of Biometeorology, 2012 or 2013.
Luedeling E. Climate change impacts on winter chill for temperate fruit and nut production: a review. Scientia Horticulturae 144, 218-229.
Luedeling E and Gassner A, 2012. Partial Least Squares Regression for analyzing walnut phenology in California. Agricultural and Forest Meteorology 158, 43-52.

**Examples**

```
require(chillR)
data(KA_weather)
data(KA_bloom)
weather<-KA_weather
weather$Tmin<-interpolate_gaps(KA_weather$Tmin)[[1]]
weather$Tmax<-interpolate_gaps(KA_weather$Tmax)[[1]]

PLS_results_path<-paste(getwd(),"/PLS_output",sep="")

PLS_results<-PLS_pheno(
    weather_data_frame=KA_weather,
    split_month=6,   #last month in same year
    bio_data_frame=KA_bloom,
    PLS_results_path=PLS_results_path)

temps<-PLS_results$weather_file


#for the chilling and heat requirement calculations, the resulting
#output files (the image and the table) should be evaluated for
#likely candidate dates for the beginnings and ends of chilling and
#forcing periods. Chilling and forcing between these dates can then
#be summarized using the calls below.

THourly<-make_hourly_temps(latitude=50.4,year_file=temps)
THours<-stack_hourly_temps(hour_file=THourly)

#a simple call to the chilling function produces values for all
#years on record between Start_JDay and End_JDay (these are Julian
#days, i.e. days of the year)

chilling(THourly=THours,Start_JDay=306,End_JDay=42)

#the results can be processed further manually. Alternatively, the
#following set of calls produces an automated evaluation of chilling
#and forcing during different periods, indicated by the Start and
#End columns in the results data.frame created below. The evaluation
#here is restricted to the years for which phenological dates were
#available (bioyears).

results<-data.frame(Start=c(306,325,325),End=c(42,42,356))

suppressWarnings(bioyears<-KA_bloom[which(!is.na(as.numeric(
 as.character(KA_bloom$pheno)))),"Year"])

incl <- function(x, table) match(x, table, nomatch = 0) > 0

for (i in 1:nrow(results))
   {tab<-chilling(THours,results[i,"Start"],results[i,"End"])
    results[i,"CH_mean"]<-round(mean(tab[which(incl(tab$End_year,
       bioyears)),"Chilling_Hours"]))
    results[i,"CH_stdev"]<-round(sd(tab[which(incl(tab$End_year,
       bioyears)),"Chilling_Hours"]))
    results[i,"CH_CV"]<-round(results[i,"CH_stdev"]/results[i,
       "CH_mean"]*1000)/10
```

```
results[i,"Utah_mean"]<-round(mean(tab[which(incl(tab$End_year,
   bioyears)),"Utah_Model"]))
results[i,"Utah_stdev"]<-round(sd(tab[which(incl(tab$End_year,
   bioyears)),"Utah_Model"]))
results[i,"Utah_CV"]<-round(results[i,"Utah_stdev"]/results[i,
   "Utah_mean"]*1000)/10
results[i,"CP_mean"]<-round(mean(tab[which(incl(tab$End_year,
   bioyears)),"Chill_portions"])*10)/10
results[i,"CP_stdev"]<-round(sd(tab[which(incl(tab$End_year,
   bioyears)),"Chill_portions"])*10)/10
results[i,"CP_CV"]<-round(results[i,"CP_stdev"]/results[i,
   "CP_mean"]*1000)/10
results[i,"GDH_mean"]<-round(mean(tab[which(incl(tab$End_year,
   bioyears)),"GDH"]))
results[i,"GDH_stdev"]<-round(sd(tab[which(incl(tab$End_year,
   bioyears)),"GDH"]))
results[i,"GDH_CV"]<-round(results[i,"GDH_stdev"]/results[i,
   "GDH_mean"]*1000)/10}
```

---

`chilling` *chilling*

---

## Description

This function calculates winter chill for temperate fruit trees according to three commonly used models: the Chilling Hours Model, the Utah Model and the Dynamic Model. It also calculates Growing Degree Days.

## Usage

```
chilling(THourly, Start_JDay, End_JDay)
```

## Arguments

| | |
|---|---|
| `THourly` | THourly is a data frame with columns Year, JDay, Hour and Temp. In this, year should be in YYYY format. JDay is the Julian Day, that is the day of the year (e.g. Jan 1 is JDay 1, Feb 1 is JDay 32), Hour is in HH format, and Temp is in degrees Celcius. |
| `Start_JDay` | This is the Julian Day at which the calculation of chill units should start. |
| `End_JDay` | This is the Julian Day at which the calculation of chill units should end. |

## Value

The function returns a data frame containing calculated chill and growing degrees for each year.

## Author(s)

Eike Luedeling

## References

Luedeling E and Brown PH, 2011. Equivalence of winter chill models for fruit and nut trees around the world. International Journal of Biometeorology 55, 411-421.

### Examples

```
require(chillR)
data(KA_weather)
data(KA_bloom)
weather<-KA_weather
weather$Tmin<-interpolate_gaps(KA_weather$Tmin)[[1]]
weather$Tmax<-interpolate_gaps(KA_weather$Tmax)[[1]]

PLS_results_path<-paste(getwd(),"/PLS_output",sep="")

PLS_results<-PLS_pheno(
    weather_data_frame=KA_weather,
    split_month=6,   #last month in same year
    bio_data_frame=KA_bloom,
    PLS_results_path=PLS_results_path)

temps<-PLS_results$weather_file


#for the chilling and heat requirement calculations, the resulting
#output files (the image and the table) should be evaluated for
#likely candidate dates for the beginnings and ends of chilling and
#forcing periods. Chilling and forcing between these dates can then
#be summarized using the calls below.

THourly<-make_hourly_temps(latitude=50.4,year_file=temps)
THours<-stack_hourly_temps(hour_file=THourly)

#a simple call to the chilling function produces values for all
#years on record between Start_JDay and End_JDay (these are Julian
#days, i.e. days of the year)

chilling(THourly=THours,Start_JDay=306,End_JDay=42)

#the results can be processed further manually. Alternatively, the
#following set of calls produces an automated evaluation of chilling
#and forcing during different periods, indicated by the Start and
#End columns in the results data.frame created below. The evaluation
#here is restricted to the years for which phenological dates were
#available (bioyears).

results<-data.frame(Start=c(306,325,325),End=c(42,42,356))

suppressWarnings(bioyears<-KA_bloom[which(!is.na(as.numeric(
 as.character(KA_bloom$pheno)))),"Year"])

incl <- function(x, table) match(x, table, nomatch = 0) > 0

for (i in 1:nrow(results))
   {tab<-chilling(THours,results[i,"Start"],results[i,"End"])
    results[i,"CH_mean"]<-round(mean(tab[which(incl(tab$End_year,
       bioyears)),"Chilling_Hours"]))
    results[i,"CH_stdev"]<-round(sd(tab[which(incl(tab$End_year,
       bioyears)),"Chilling_Hours"]))
    results[i,"CH_CV"]<-round(results[i,"CH_stdev"]/results[i,
       "CH_mean"]*1000)/10
```

```
    results[i,"Utah_mean"]<-round(mean(tab[which(incl(tab$End_year,
       bioyears)),"Utah_Model"]))
    results[i,"Utah_stdev"]<-round(sd(tab[which(incl(tab$End_year,
       bioyears)),"Utah_Model"]))
    results[i,"Utah_CV"]<-round(results[i,"Utah_stdev"]/results[i,
       "Utah_mean"]*1000)/10
    results[i,"CP_mean"]<-round(mean(tab[which(incl(tab$End_year,
       bioyears)),"Chill_portions"])*10)/10
    results[i,"CP_stdev"]<-round(sd(tab[which(incl(tab$End_year,
       bioyears)),"Chill_portions"])*10)/10
    results[i,"CP_CV"]<-round(results[i,"CP_stdev"]/results[i,
       "CP_mean"]*1000)/10
    results[i,"GDH_mean"]<-round(mean(tab[which(incl(tab$End_year,
       bioyears)),"GDH"]))
    results[i,"GDH_stdev"]<-round(sd(tab[which(incl(tab$End_year,
       bioyears)),"GDH"]))
    results[i,"GDH_CV"]<-round(results[i,"GDH_stdev"]/results[i,
       "GDH_mean"]*1000)/10}
```

---

`interpolate_gaps` *interpolate_gaps*

---

### Description

This function takes a vector of values and closes all gaps in the list (NA values) by linear interpolation.

### Usage

```
interpolate_gaps(x)
```

### Arguments

| | |
|---|---|
| `x` | x is a vector of numeric values, which can contain NA values and non-numeric values, which are then interpreted as missing values. |

### Value

The function returns a list of two vectors interp and miss. interp is similar to vector x where x had values, but all missing values from x are closed by linear interpolation. miss contains positions of all values in vector x that were interpolated.

### Author(s)

Eike Luedeling

### Examples

```
require(chillR)
data(KA_weather)
data(KA_bloom)
weather<-KA_weather
weather$Tmin<-interpolate_gaps(KA_weather$Tmin)[[1]]
```

```
weather$Tmax<-interpolate_gaps(KA_weather$Tmax)[[1]]
```

---

`KA_bloom` *Cherry bloom data*

---

## Description

Bloom data of cv. Schneiders' cherries at Klein-Altendorf, Germany, recorded at the experimental station of the University of Bonn between 1984 and 2008.

## Usage

```
data(KA_bloom)
```

## Format

A data frame with 25 observations on the following 2 variables.

`Year` observation year: a numeric vector

`pheno` Julian date of cherry bloom in the respective year: this is numeric but stored as a factor, because missing data is coded by a string

## Source

Data provided by Michael Blanke and Achim Kunz, University of Bonn, Germany

## References

Luedeling E, Kunz A and Blanke M. Identification of chilling and heat requirements of cherry trees - a statistical approach. International Journal of Biometeorology, 2012 or 2013.

## Examples

```
data(KA_bloom)
```

---

`KA_weather` *Temperature records from Klein-Altendorf*

---

## Description

Minimum and maximum daily temperatures collected at the experimental station of the University of Bonn at Klein-Altendorf, Germany. Some records are missing, but functions for gap interpolation are provided in this package.

## Usage

```
data(KA_weather)
```

## Format

A data frame with 19144 observations on the following 5 variables.

`Year` observation year: a numeric vector

`Month` observation month: a numeric vector

`Day` observation day: a numeric vector

`Tmax` daily maximum temperature (C): numeric vector, but stored as a factor, because missing values are coded by a character string

`Tmin` daily minimum temperature (C): numeric vector, but stored as a factor, because missing values are coded by a character string

## Source

Data provided by Michael Blanke and Achim Kunz, University of Bonn, Germany

## References

Luedeling E, Kunz A and Blanke M. Identification of chilling and heat requirements of cherry trees - a statistical approach. International Journal of Biometeorology, 2012 or 2013.

## Examples

```
data(KA_weather)
```

---

# `make_hourly_temps` *make_hourly_temps*

---

## Description

This function makes hourly temperatures from daily minimuma and maximum temperatures.

## Usage

```
make_hourly_temps(latitude, year_file)
```

## Arguments

| | |
|---|---|
| `latitude` | The latitude of the location, for which hourly temperatures are to be modeled. |
| `year_file` | A data.frame without gaps of daily minimum and maximum temperatures. The data.frame can have additional columns, but must include columns Tmin and Tmax. |

## Value

The outut is a data.frame that is similar to the input data.frame, but has 24 additional columns containing modeled hourly temperatures

## Author(s)

Eike Luedeling

### References

Linvill DE (1990) Calculating chilling hours and chill units from daily maximum and minimum temperature observations. Hortscience 25, 14-16.
Spencer JW (1971) Fourier series representation of the position of the Sun. Search 2, 172.
Almorox J, Hontoria C, Benito M (2005) Statistical validation of daylength definitions for estimation of global solar radiation in Toledo, Spain. Energy Convers Manage 46, 1465-1471.
Luedeling E and Brown PH, 2011. Equivalence of winter chill models for fruit and nut trees around the world. International Journal of Biometeorology 55, 411-421.

### Examples

```
require(chillR)
data(KA_weather)
data(KA_bloom)
weather<-KA_weather
weather$Tmin<-interpolate_gaps(KA_weather$Tmin)[[1]]
weather$Tmax<-interpolate_gaps(KA_weather$Tmax)[[1]]

PLS_results_path<-paste(getwd(),"/PLS_output",sep="")

PLS_results<-PLS_pheno(
    weather_data_frame=KA_weather,
    split_month=6,   #last month in same year
    bio_data_frame=KA_bloom,
    PLS_results_path=PLS_results_path)

temps<-PLS_results$weather_file


#for the chilling and heat requirement calculations, the resulting
#output files (the image and the table) should be evaluated for likely
#candidate dates for the beginnings and ends of chilling and forcing
#periods. Chilling and forcing between these dates can then be
#summarized using the calls below.

THourly<-make_hourly_temps(latitude=50.4,year_file=temps)
```

---

PLS_pheno *PLS_pheno*

---

### Description

This function performs a PLS regression analysis, in which yearly observations of cherry bloom are correlated with a running mean of mean daily temperatures of a 12-month period. Side effects are outputs of two tables and one image in the PLS_results_path directory. This function includes interpolation of missing daily temperature data, using the function interpolate_gaps from this package.

### Usage

```
PLS_pheno(weather_data_frame,bio_data_frame, split_month,
          PLS_results_path, runn_mean)
```

## Arguments

`weather_data_frame`
: A data.frame of weather data, including the columns Tmin and Tmax for minimum and maximum daily temperatures. It must also include columns Year, Month and Day to indicate the date of temperature observations.

`bio_data_frame`
: This is a data.frame of phenological observations, such as the bloom date of cherry trees. It must consist of two columns named Year and pheno.

`split_month`
: This is the last month of the year (indicated by a number), for which temperature data is correlated with phenological data for the respective year. Temperatures for all later months are assigned to the previous year, so that a total of one full year is used as independent variables in the PLS regression.

`PLS_results_path`
: The path where PLS outputs (two tables and one image) are saved.

`runn_mean`
: Unprocessed daily mean temperatures are typically too noisy to produce interpretable PLS outputs. This number (which should be odd) processes running means of the daily mean temperatures, which are then used as inputs into the PLS regression.

## Value

The result is a list of two data.frames: weather_file: contains all inputs into the PLS analysis PLS_output: contains the results from the PLS regression Both data.frames are also saved as .csv tables in the PLS_results_path folder.

## Author(s)

Eike Luedeling

## References

Luedeling E and Gassner A, 2012. Partial Least Squares Regression for analyzing walnut phenology in California. Agricultural and Forest Meteorology 158, 43-52. Luedeling E, Kunz A and Blanke M. Identification of chilling and heat requirements of cherry trees - a statistical approach. International Journal of Biometeorology, 2012 or 2013.

## Examples

```
require(chillR)
data(KA_weather)
data(KA_bloom)
weather<-KA_weather
weather$Tmin<-interpolate_gaps(KA_weather$Tmin)[[1]]
weather$Tmax<-interpolate_gaps(KA_weather$Tmax)[[1]]

PLS_results_path<-paste(getwd(),"/PLS_output",sep="")

PLS_results<-PLS_pheno(
    weather_data_frame=KA_weather,
    split_month=6,   #last month in same year
    bio_data_frame=KA_bloom,
    PLS_results_path=PLS_results_path)
```

```
temps<-PLS_results$weather_file
```

---

`stack_hourly_temps` *stack_hourly_temps*

---

### Description

In the output of the function make_hourly_temps, hourly temperatures are listed in columns per day. This function takes these hourly temperatures and stacks them, so that the result is a long list of hours with the respective hourly temperatures.

### Usage

```
stack_hourly_temps(hour_file)
```

### Arguments

| | |
|---|---|
| `hour_file` | this is the output of the make_hourly_temps function |

### Value

The output is a data.frame, with all columns of the input data.frame, except the Hourly temperature columns. Additional columns are Hour (as integer) and Temp (the hourly temperature).

### Author(s)

Eike Luedeling

### Examples

```
require(chillR)
data(KA_weather)
data(KA_bloom)
weather<-KA_weather
weather$Tmin<-interpolate_gaps(KA_weather$Tmin)[[1]]
weather$Tmax<-interpolate_gaps(KA_weather$Tmax)[[1]]

PLS_results_path<-paste(getwd(),"/PLS_output",sep="")

PLS_results<-PLS_pheno(
    weather_data_frame=KA_weather,
    split_month=6,   #last month in same year
    bio_data_frame=KA_bloom,
    PLS_results_path=PLS_results_path)

temps<-PLS_results$weather_file


#for the chilling and heat requirement calculations, the resulting
#output files (the image and the table) should be evaluated for likely
#candidate dates for the beginnings and ends of chilling and forcing
#periods. Chilling and forcing between these dates can then be
#summarized using the calls below.
```

```
THourly<-make_hourly_temps(latitude=50.4,year_file=temps)
THours<-stack_hourly_temps(hour_file=THourly)
```

# Index

∗Topic **chillR**
    chilling, 4
    interpolate_gaps, 6
    make_hourly_temps, 8
    PLS_pheno, 9
    stack_hourly_temps, 11
∗Topic **datasets**
    KA_bloom, 7
    KA_weather, 7
∗Topic **package**
    chillR-package, 2

chilling, 4
chillR *(chillR-package)*, 2
chillR-package, 2

interpolate_gaps, 6

KA_bloom, 7
KA_weather, 7

make_hourly_temps, 8

PLS_pheno, 9

stack_hourly_temps, 11
