# Supplementary material for: Identification of chilling and heat requirements of cherry trees—a statistical approach
Source: Int J Biometeorol. 2012 Oct 6;57(5):679–89. doi: 10.1007/s00484-012-0594-y (PMC3745618; doi:10.1007/s00484-012-0594-y)
Supplement: Supplementary file 2 — (PDF 282 kb) [file 484_2012_594_MOESM2_ESM.pdf]

Running the PLS procedure in the chillR package for determining chilling and heat requirements of tree crops

by Eike Luedeling, World Agroforestry Centre

August 2012

Installation of the chillR package

Install R (from http://www.R-project.org/)
Open R and install the chillR package. The command for doing this is as follows:

```
install.packages("D:/work/chillR/chillR_0.5.tar.gz",repos=NULL,type='source')
```

The path "D:/work/chillR/chillR_0.5.tar.gz" should be replaced by the path leading to the package source.

Once this command has been executed successfully, the R command 'require(chillR)' should load the package. It is also necessary to install the pls package from one of the CRAN mirrors (from the R console, select 'Packages→Install package(s)' from the menu. From the next list, select an R mirror near you, then select the package 'pls' from the package list).

Running the PLS analysis with the chillR sample data

The following example script will produce PLS results similar to those reported in Luedeling et al. (2012 or 2013, though based on a shorter dataset). Results are saved in the current working directory, but that can easily be changed by replacing the PLS_results_path command below with:
PLS_results_path<-"X:/[directory_path]/[file_name_start]":

```
require(chillR)
data(KA_weather)
data(KA_bloom)
weather<-KA_weather
weather$Tmin<-interpolate_gaps(KA_weather$Tmin)[[1]]
weather$Tmax<-interpolate_gaps(KA_weather$Tmax)[[1]]

PLS_results_path<-paste(getwd(),"/PLS_output",sep="")

PLS_results<-PLS_pheno(weather_data_frame=KA_weather,split_month=6,
   bio_data_frame=KA_bloom,PLS_results_path=PLS_results_path)
```

The above script opens the datasets provided in the package (abbreviated version of the ones used in Luedeling et al. (2012 or 2013)), and processes them into a PLS results image and a table of the respective model coefficients and VIP scores. During data preparation, gaps in the daily records of

minimum and maximum temperatures are filled by linear interpolation. All outputs are saved in the indicated location (the working directory, if you don't change the PLS_results_path).

Evaluation of the PLS outputs is best done visually based on the PLS figure. Exact dates can then be extracted from the results table. In the figure, long periods of positive model coefficients, especially if they are colored in green and blue in the various panels of the figure, indicate periods, during which high temperatures are associated with later bloom dates (or timings of other phenological stages). These phases are likely to correspond to chill accumulation. In contrast, periods of negative model coefficients, especially when shown in blue and red, are phases with a negative relationship between warm temperatures and phenological dates. These are likely the forcing phases. The beginning and end of interesting phases should be determined by the user. Chill and forcing during these phases can then be determined using the following R commands. Here it is assumed that the results of the PLS_pheno function are stored in the variable PLS_results (as shown in the series of R commands shown above) and that the latitude of the study location is 50.4 degrees north (can be adjusted):

```
temps<-PLS_results$weather_file
THourly<-make_hourly_temps(latitude=50.4,year_file=temps)
THours<-stack_hourly_temps(hour_file=THourly)

chilling(THourly=THours,Start_JDay=306,End_JDay=42)
```

The first three lines above prepare the weather record for the analysis, including production of hourly temperature records from daily values using idealized daily temperature curves. Such hourly records are needed for calculating chill according to commonly used models.
The forth line calculates chill and forcing for each year on record, as accumulated between the indicated dates. These are required as Julian Days (days of the year; e.g. Jan 12th is JDay 12; Nov 1st is JDay 305). The result is a table with chill totals in Chilling Hours, Utah Chill Units and Chill Portions, as well as Growing Degree Days, for each year of the weather record. These are given as R output. They can also be written to a .csv table using the following command:

```
write.csv(chilling(THourly=THours,Start_JDay=306,End_JDay=42,”X:/[directory]/[file_name].csv”)
```

The last part of the above statement is the full path name to the .csv file to be written. Start_JDay and End_JDay can also be adjusted.
The results can be processed further manually. Alternatively, the following set of calls produces an automated evaluation of chilling and forcing during different periods, indicated by the Start and End columns in the results data.frame created below. The evaluation here is restricted to the years for which phenological dates were available (bioyears). First, start and end days of interesting periods should be entered into the following lists, with all start dates in the Start vector, and all End dates in the End vector:

```
results<-data.frame(Start=c(306,325,325,356,23,43,43),End=c(42,42,356,7,42,93,108))
```

The remaining calls determine the years for which phenological data are available and then call the chilling function multiple times to calculate mean chill/forcing and coefficients of variation for each metric over all of these years. The last command below writes all results into a user-specified location as a .csv file (path must be adjusted).

```
suppressWarnings(bioyears<-
KA_bloom[which(!is.na(as.numeric(as.character(KA_bloom$pheno)))),"Year"])
```

```
for (i in 1:nrow(results))
  {tab<-chilling(THours,results[i,"Start"],results[i,"End"])
   results[i,"CH_mean"]<-round(mean(tab[which(tab$End_year %in% bioyears),"Chilling_Hours"]))
   results[i,"CH_stdev"]<-round(sd(tab[which(tab$End_year %in% bioyears),"Chilling_Hours"]))
   results[i,"CH_CV"]<-round(results[i,"CH_stdev"]/results[i,"CH_mean"]*1000)/10
   results[i,"Utah_mean"]<-round(mean(tab[which(tab$End_year %in% bioyears),"Utah_Model"]))
   results[i,"Utah_stdev"]<-round(sd(tab[which(tab$End_year %in% bioyears),"Utah_Model"]))
   results[i,"Utah_CV"]<-round(results[i,"Utah_stdev"]/results[i,"Utah_mean"]*1000)/10
   results[i,"CP_mean"]<-round(mean(tab[which(tab$End_year %in% bioyears),"Chill_portions"])*10)/10
   results[i,"CP_stdev"]<-round(sd(tab[which(tab$End_year %in% bioyears),"Chill_portions"])*10)/10
   results[i,"CP_CV"]<-round(results[i,"CP_stdev"]/results[i,"CP_mean"]*1000)/10
   results[i,"GDH_mean"]<-round(mean(tab[which(tab$End_year %in% bioyears),"GDH"]))
   results[i,"GDH_stdev"]<-round(sd(tab[which(tab$End_year %in% bioyears),"GDH"]))
   results[i,"GDH_CV"]<-round(results[i,"GDH_stdev"]/results[i,"GDH_mean"]*1000)/10}

write.csv(results,"X:/[directory]/[file_name].csv")
```

Some steps of the procedure can take some time, especially on slow computers, because the hourly temperature records can get quite long, especially when many years of weather records are available.

Customizing the process for new datasets

In principle, all steps of the analysis described above should work for any phenology/weather dataset. The only change required is the definition of the input files, which should take the following form:

```
KA_weather<-read.csv("X:/[directory]/[weather_file].csv ")
KA_bloom<-read.csv("X:/[directory]/[pheno_file].csv")
```

(instead of data(KA_weather) and data(KA_bloom))

The two input tables (for weather and phenology) should be provided as .csv files and organized as follows:

Weather file:

Year	Month	Day	Tmax	Tmin
1951	1	1	15	3.9
1951	1	2	10	0.6
1951	1	3	10	3.3
1951	1	4	14.4	7.8
1951	1	5	Missing	4.4
1951	1	6	13.3	3.9
1951	1	7	11.1	Missing
1951	1	8	10	2.8

For missing data, any character string or NA is recognized.

Phenology file:

Year	pheno
1953	75
1954	92
1955	Missing
1956	79

The column pheno contains the Julian Date of the phenological event for each year.
Note that in both tables the column names must be as specified in these examples, including correct capitalization. Note also that all paths were written as they should appear in a Windows system, the only system for which the package has been tested.

More help

In R, help on any function can be obtained by typing ?[function], e.g. ?chilling or ?PLS_pheno. A pdf version of all help files is also included in the supplementary materials to Luedeling et al. (2012 or 2013), alongside this tutorial.
